# Functionalised Hybrid Collagen-Elastin for Acellular Cutaneous Substitute Applications

**DOI:** 10.3390/polym15081929

**Published:** 2023-04-18

**Authors:** Nurkhuzaiah Kamaruzaman, Mh Busra Fauzi, Yasuhiko Tabata, Salma Mohamad Yusop

**Affiliations:** 1 Department of Food Sciences, Faculty of Science and Technology, Universiti Kebangsaan Malaysia, Bangi 43600, Selangor, Malaysia; 2 Centre for Tissue Engineering and Regenerative Medicine, Faculty of Medicine, Universiti Kebangsaan Malaysia, Kuala Lumpur 56000, Selangor, Malaysia; 3 Laboratory of Biomaterials, Department of Regeneration Science and Engineering, Institute for Life and Medical Sciences (LiMe), Kyoto University, 53 Kawara-cho Shogoin, Sakyo-Ku, Kyoto 606-8507, Japan

**Keywords:** elastin, collagen, hybrid bioscaffold, tissue engineering, acellular skin substitute

## Abstract

Wound contracture, which commonly happens after wound healing, may lead to physical distortion, including skin constriction. Therefore, the combination of collagen and elastin as the most abundant extracellular matrix (ECM) skin matrices may provide the best candidate biomaterials for cutaneous wound injury. This study aimed to develop a hybrid scaffold containing green natural resources (ovine tendon collagen type-I and poultry-based elastin) for skin tissue engineering. Briefly, freeze-drying was used to create the hybrid scaffolds, which were then crosslinked with 0.1% (w/v) genipin (GNP). Next, the physical characteristics (pore size, porosity, swelling ratio, biodegradability and mechanical strength) of the microstructure were assessed. Energy dispersive X-ray spectroscopy (EDX) and Fourier transform infrared (FTIR) spectrophotometry were used for the chemical analysis. The findings showed a uniform and interconnected porous structure with acceptable porosity (>60%) and high-water uptake capacity (>1200%), with pore sizes ranging between 127 ± 22 and 245 ± 35 µm. The biodegradation rate of the fabricated scaffold containing 5% elastin was lower (<0.043 mg/h) compared to the control scaffold (collagen only; 0.085 mg/h). Further analysis with EDX identified the main elements of the scaffold: it contained carbon (C) 59.06 ± 1.36–70.66 ± 2.89%, nitrogen (N) 6.02 ± 0.20–7.09 ± 0.69% and oxygen (O) 23.79 ± 0.65–32.93 ± 0.98%. FTIR analysis revealed that collagen and elastin remained in the scaffold and exhibited similar functional amides (amide A: 3316 cm^−1^, amide B: 2932 cm^−1^, amide I: 1649 cm^−1^, amide II: 1549 cm^−1^ and amide III: 1233 cm^−1^). The combination of elastin and collagen also produced a positive effect via increased Young’s modulus values. No toxic effect was identified, and the hybrid scaffolds significantly supported human skin cell attachment and viability. In conclusion, the fabricated hybrid scaffolds demonstrated optimum physicochemical and mechanical properties and may potentially be used as an acellular skin substitute in wound management.

## 1. Introduction

Injuries to the skin, including chronic wounds, ulcers and burns, are among the most prevalent and significant issues in human health care, in terms of both social and economic impact, and they cost the US more than $28 billion per year [1]. Skin allografts, autografts and xenografts can now be used to partially treat various skin injuries. However, elastin is improperly regenerated and disorganised in scar tissue following severe burns. As a result, an intact elastic fibre network is absent after a cutaneous injury, contributing to scars’ deteriorating physical properties compared with those of intact skin [2,3]. Clinicians and researchers are realising that after a severe burn, the elastic fibre network needs to be rebuilt for wounds to heal in a way that is both functional and aesthetically pleasing. This makes elastin an important component of wound healing.

As the second most abundant protein in the human body and one of the major components of extracellular matrix (ECM) in the skin, elastin is believed to play vital roles in both cell signalling and structural components. Thus, its presence may lessen the severe skin contraction and scarring linked to burn injuries while also promoting wound healing [4]. Elastin may produce these effects by altering the differentiation of fibroblasts into contractile myofibroblasts [5,6,7] and promoting new elastin expression in a healing wound [8,9,10]. In addition, elastin has been shown to enhance angiogenesis [11,12]. Even though elastin has demonstrated positive effects on dermal function and regeneration, its insolubility drawbacks in three-dimensional bioscaffold construction remain underexplored. However, given that both soluble elastin and the recombinant elastin precursor tropoelastin are currently available, elastin-based peptides now permit a greater and more effective use of this protein in the development of advanced wound care [4,13].

As the most abundant ECM protein, collagen is in high demand in the tissue engineering, regenerative medicine and cosmetics industries. Due to its excellent biological properties (such as low immunogenicity, porous structure, good permeability and biodegradability), it is widely utilised in tissue engineering [14,15]. However, collagen scaffolds often have limited strength and lack elasticity. Therefore, a dual combination of elastin and collagen may overcome the collagen scaffold’s disadvantages of poor structural stability and mechanical properties [2,16]. In addition, multifunctional smart biomaterial is currently being explored in tissue engineering as an advanced technology to promote the regeneration of skin that resembles native skin. The use of natural polymers from green resources contributes to larger socio-economic benefits for countries worldwide. Therefore, combining a poultry-based elastin scaffold with another natural biomaterial to replicate the ECM microenvironment structurally, mechanically, physiologically and functionally will be beneficial. Biomaterials derived from natural elastin are commonly extracted from animal tissue, including bovine, porcine, poultry and marine sources [17,18,19,20]. Chicken is considered an essential source of protein; its consumption has been quite high in recent years, possibly because there is not much religious prohibition against chicken consumption compared to that of other meats. Hence, poultry-based elastin represents a new generation of elastin biomaterial widely accepted by all religious communities and cultures.

In our previous studies, elastin peptide from chicken skin has been successfully extracted and characterised [17,21,22,23]. However, its potential as a biomaterial for skin tissue engineering has not been explored. Therefore, in this study we developed a hybrid scaffold composed of collagen and elastin, and then explored its physicochemical and mechanical characteristics (in the form of a 3D-bioscaffold) to assess its potential for future use in skin tissue engineering. In addition, both its cellular biocompatibility and its toxicity towards human skin cells were evaluated.

## 2. Materials and Methods

This research was approved by the Research Ethics Committee Universiti Kebangsaan Malaysia (UKM PPI/111/8/JEP-2020-453).

### 2.1. Extraction and Purification of Elastin from Broiler Skin

The method reported in our previous studies was used to extract elastin from broiler skin [23,24]. After thawing, the broiler skin was manually defatted. The skin was then immersed in NaCl overnight in a cold room. The homogenate was centrifuged at 11,000 rpm (Eppendorf 5804R, Hamburg, Germany). Consequently, the pellet was washed and underwent further defatting steps. The treated sample was then suspended in NaOH and heated with constant shaking. The residues of NaOH-insoluble material were washed several times in water and lyophilised before further analyses. Subsequently, treatment with acid was done at 100 °C to enable water-soluble elastin formation before freeze-drying.

### 2.2. Extraction and Purification of Collagen Type-I from Ovine Tendon

Collagen extraction and purification processes were conducted according to the method given in a previous study [15]. The crude tendon was cleaned of fur, skin, fascia and muscle tissues. The tendon was then dissolved in 0.35 M acetic acid at 4 °C for 24–48 h until it was swollen. Subsequently, the tendon was blended and centrifuged (5000 rpm, 5 min, 4 °C). The supernatant was mixed with sodium chloride (0.05 g/mL); (Sigma-Aldrich, St. Louis, MO, USA) and kept at 4 °C for 24 h. After that, it was centrifuged at 5000 rpm for 5 min. The collagen pellet was then run through a dialysis tube (molecular weight cut-off = 14 kDa) (Sigma-Aldrich, St. Louis, MO, USA) for 72 h, with distilled water as the dialysis buffer being changed every 12 h. Collagen that had been dialysed was freeze-dried for 72 h and then re-dissolved in 0.35 M acetic acid to make a collagen concentration of 15 mg/mL for further use.

### 2.3. Fabrication of Collagen-Elastin (Col/Elas) Scaffolds

Col/Elas scaffolds were fabricated by combining different percentages of elastin with the collagen solution. Briefly, the 15 mg/mL collagen solution was first pipetted into a centrifuge tube. Then, 2–5% of elastin powder was weighed and mixed with the collagen solution using a mixer. After that, the solution was pipetted into the desired mould and pre-frozen at −80 °C for 6 h, and then freeze-dried for 24–48 h. For the control group, the scaffolds were made of collagen only (Col). The fabricated scaffolds were subsequently cross-linked with 0.1% genipin solution (Wako, Japan) at 25 °C for 6 h. After 6 h of immersion, the genipin solution was removed, and then the scaffolds were pre-frozen and freeze-dried for 72 h. The scaffolds were as follows: Col (collagen solution only), Col/Elas-2 (2% elastin added), Col/Elas-3 (3% elastin added), Col/Elas-4 (4% elastin added) and Col/Elas-5 (5% elastin added).

### 2.4. Degree of Crosslinking

Approximately 10 mg of the test sample was weighed and heated with 200 µL of 10× ninhydrin solution (Sigma-Aldrich, St. Louis, MO, USA) for 20 min. After the test solution was cooled to room temperature and diluted in 200 µL of 95% ethanol, the optical absorbance of the solution was measured using a UV-visible spectrophotometer at 570 nm with glycine (Gly) at various concentrations (0.006, 0.0125, 0.025, 0.05 and 0.1 mg/mL) as the standard. The amount of free amino groups in hybrid Col/Elas scaffolds before (C_i_) and after (C_f_) crosslinking is proportional to the optical absorbance of the solution. The degree of crosslinking of the scaffolds was calculated using the equation below:

(1) $$\mathrm{Degree}\;\mathrm{of}\;\mathrm{crosslinking}\;(\%)\;=\frac{C_{i}-C_{f}}{C_{i}}\times 100\;$$

### 2.5. Swelling Ratio

First, the initial dry weight of the scaffolds was determined (W_0_). The samples were then immersed in 2 mL of PBS (Sigma-Aldrich, St. Louis, MO, USA) for 24 h at room temperature. The excess water was removed using filter paper and the final weight of the sample (W_1_) was recorded. The swelling ratio was calculated as follows:

(2) $$W_{s}\;(\%)=\frac{W_{1}-W_{0}}{W_{0}}\times 100$$

### 2.6. Porosity

The initial dried weight (W_1_) of each scaffolds was measured. The scaffolds were then immersed in absolute ethanol 99.5% (w/w) (Systerm, Shah Alam, Malaysia) overnight. Next, the scaffolds were removed from the ethanol and blotted with filter paper to remove excess ethanol and immediately weighed (W_2_). The porosity of the scaffolds was measured using the liquid replacement method [25]. Porosity (%) was calculated using the equation below:

(3) $$\mathrm{Porosity}\;\left(\%\right)=\frac{M_{2}-M_{1}}{\rho V}\times 100$$

where V denotes the volume of the scaffolds and ρ refers to the density of absolute ethanol (0.789 g/mL).

### 2.7. Biodegradation

The weight loss of the scaffolds after a certain period was used to calculate the biodegradation rate of the scaffolds. The samples’ initial weight (dry) was determined (W_0_), and they were then immersed in 2 mL of 0.0006% (w/v) collagenase (Worthington, Lakewood, NJ, USA), which was followed by a 37 °C incubation. The collagenase was removed from the samples after 24 h. After that, the samples were gently rinsed with distilled water before being freeze-dried. Following that, the weight (W_t_) was recorded. The weight loss rate of the scaffolds was calculated using the equation below:

(4) $$\mathrm{Biodegradation}\;\mathrm{rate}\left(\mathrm{mg}/h\right)=\frac{W_{0}-W_{t}}{h}$$

where W_0_ is the dry weight, W_t_ is the final weight in a dry form and h is the time taken.

### 2.8. Chemical Characterisation of Scaffolds

Chemical characterisation of the Col/Elas scaffolds was carried out using EDX and FTIR. Scaffolds were scanned for EDX using an INCA Energy 2000 microscope (Oxford Instruments, Abingdon, UK) to characterise the main elements containing carbon, nitrogen and oxygen compositions. XRD was used to obtain atomic-scale structural information about the scaffolds. FTIR spectra were obtained using a Perkin Elmer Spectrum 400 FTIR Spectrometer with wavenumbers ranging from 400 to 4000 cm^−1^ (PerkinElmer, Waltham, MA, USA).

### 2.9. Microstructure of Scaffolds

Scaffolds were prepared and visualised under the Leo 1450 VP-SEM (Zeiss, Dublin, CA, USA). The freeze-dried scaffolds were placed on the mounting platform using two-sided carbon tape and then coated with gold using a Polaron SC 7680 sputter-coating device (Polaron, London, UK) for 60 s. Subsequently, the samples were introduced into the specimen chamber and their surface morphology was examined at a 20 kV accelerating voltage. The scaffolds were viewed at 100× magnification.

### 2.10. Tensile Strength

Several 3 cm^2^ pieces of scaffolds were cut and attached to an Instron 8874 (Instron, Norwood, MA, USA) using a clamp at both ends. A 50 N load transducer at a crosshead velocity of 0.05 mm/min was used to evaluate their mechanical strength.

### 2.11. Human Skin Cell Harvest and Culture

Redundant skin samples were collected from all consenting healthy patients who underwent abdominoplasty or face-lift surgery. Briefly, a 3 cm^2^ skin sample was cleaned of unwanted fragments such as fat, hair and debris, then minced into small pieces (approximately 2 mm^2^). The skin was then digested with 0.6% Collagenase Type-I (Worthington, Lakewood, NJ, USA) for 4 h in a 37 °C incubator shaker, followed by cell dissociation with 0.05% Trypsin-EDTA (Gibco, Carlsbad, CA, USA) for 15 min. Digested skin containing both keratinocytes and fibroblasts was then resuspended in co-culture medium (an equivalent mixture of keratinocyte growth medium and fibroblast growth medium). Every 2–3 days, the waste medium was changed.

Following the protocol established in previous studies [15], fibroblasts were separated from co-cultured keratinocytes using differential trypsinisation after reaching 70–80% confluence. HDF at passage 2–3 were seeded at a density of 5000 cells/cm^2^ onto the scaffold. Cell attachment was measured after 24 h of culture. The samples were gently washed with Dulbecco’s Phosphate Buffered Saline (DPBS) (Sigma, Livonia, MI, USA). After that, the solution was transferred into a tube and centrifuged. The cells in DPBS were then counted using a hemocytometer and 0.4% trypan blue solution (Sigma). The following formula was used to calculate cell attachment:

(5) $$\mathrm{Cell}\;\mathrm{attachment}\;(\%)=\frac{I_{c}-N_{c}}{I_{c}}$$

where I_c_ is the initial cell seeding and N_c_ is the number of cells in DPBS.

### 2.12. Live and Dead Assay

After 24 h of seeding HDF on scaffolds, the scaffolds were stained using Invitrogen’s LIVE/DEAD^®^ Viability/Cytotoxicity Kit for mammalian cells in accordance with the manufacturer’s instructions. A working solution containing 2 µM calcein AM and 4 µM ethidium homodimer 1-red (EthD-1) in F-12: Dulbecco’s Modified Eagle Medium (1:1; FD; Gibco) was prepared. Cells were washed with DPBS and then incubated with the working solution. After 30 min, the cells were examined using a Nikon A1R confocal microscope (Nikon, Tokyo, Japan).

### 2.13. Cell Morphology

Morphological features of the cells on scaffolds were examined using FESEM. Scaffolds seeded with the cells were firstly fixed with 3% glutaraldehyde for 24 h before being subjected to dehydration with alcohol at different percentages (50%, 75% and 95%) for 10 min each. Then, all samples were sent for critical point drying (CPD) before viewing their morphology using LEO 1450 VP-SEM (Zeiss, Oberkochen, Germany) at 500× magnification.

### 2.14. Cell Viability

The viability of HDF cells on the scaffolds at 1, 5, and 7 days was analysed using the MTT assay kit according to the manufacturer’s recommendations. In each well, 100 µL of fresh medium was added, followed by 10 µL of 12 mM MTT reagent; this was then incubated for 4 h at 37 °C. The dissolution reagent was prepared by adding 10 mL of 0.01 M HCl to one tube containing 1.0 g of SDS. Then, 100 µL of dissolution reagent was added to the solution and incubated for 4 h at 37 °C. The absorbance was read at 565 nm. Cell viability was calculated as follows:

(6) $$\mathrm{Cell}\;\mathrm{viability}\;(\%)=\frac{A_{s}}{A_{c}}\times 100$$

where A_s_ is the absorbance of the sample and A_c_ is the absorbance of the control sample.

### 2.15. Statistical Analysis

The data were analysed with GraphPad Prism 8.0 (Graph Pad Software, Inc., San Diego, CA, USA). To compare the control with multiple groups, a one-way analysis of variance (ANOVA) was used, followed by a post-hoc Tukey test. Results were expressed as means and standard deviations. A difference was considered significant when the *p*-value was < 0.05.

## 3. Results

### 3.1. Physical Characterisation of Hybrid Scaffold

**Figure 1:**
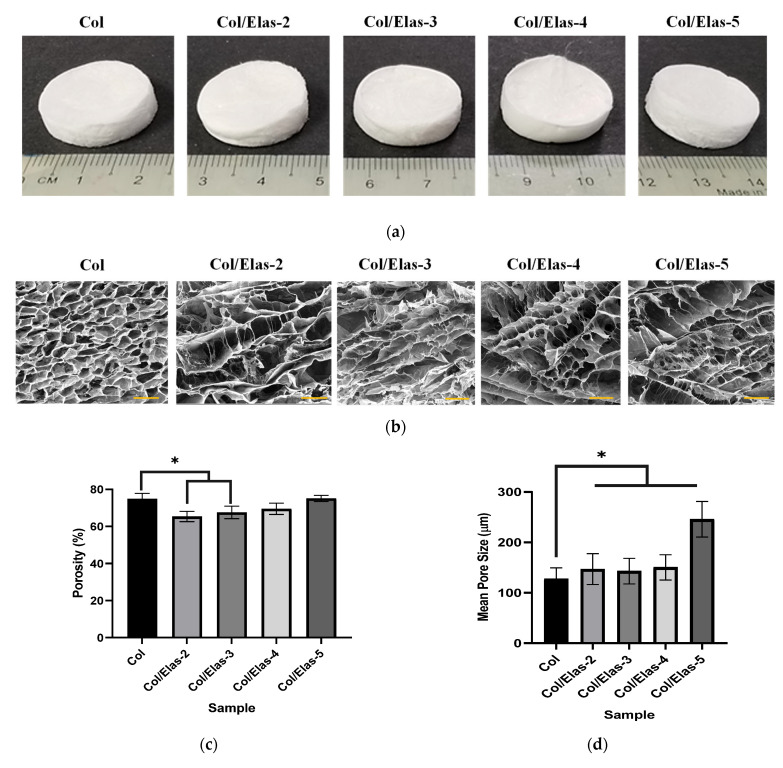
(**a**) Gross appearance of the genipin-crosslinked scaffolds after crosslinking at 25 °C. (**b**) The cross-sectional FESEM images of the scaffolds at 100× magnification (Scale bar = 200 µm). (**c**) Measured porosity of Col/Elas scaffolds. (**d**) Mean pore sizes of Col/Elas scaffolds. (*) represents a significant difference (*p* < 0.05; *n* = 3) between the scaffolds.

The gross appearances of the Col and hybrid Col/Elas scaffolds are illustrated in Figure 1a. All the crosslinked scaffolds showed a characteristically opaque structure and were slightly yellowish. The cross-sectional FESEM images of these scaffolds are given in Figure 1b. According to the morphology of the samples, all scaffolds had interconnected pores with an average pore size ranging from 100 µm to 245 µm. The pore size was also uniformly and normally distributed across the samples.

The porosity of the samples is shown in Figure 1c. Col showed a significantly higher porosity (*p* < 0.05) with a value of 74.83% ± 2.94 compared to Col/Elas-2 and Col/Elas-3, but it was not significantly different from Col/Elas-4 or Col/Elas-5. Despite that, the porosity of the treated groups increased as elastin percentage increased.

According to the data, the average pore sizes of the Col/Elas-2, Col/Elas-3, Col/Elas-4 and Col/Elas-5 were 147 ± 31 µm, 143 ± 25 µm, 150 ± 25 µm and 245 ± 35 µm, respectively (Figure 1d). These values were significantly higher (*p* < 0.05) than that of Col, which was 127 ± 22 µm. Therefore, by varying the collagen/elastin ratio, the pore size of the scaffold could be controlled: the higher the elastin percentage, the bigger the pore size.

**Figure 2:**
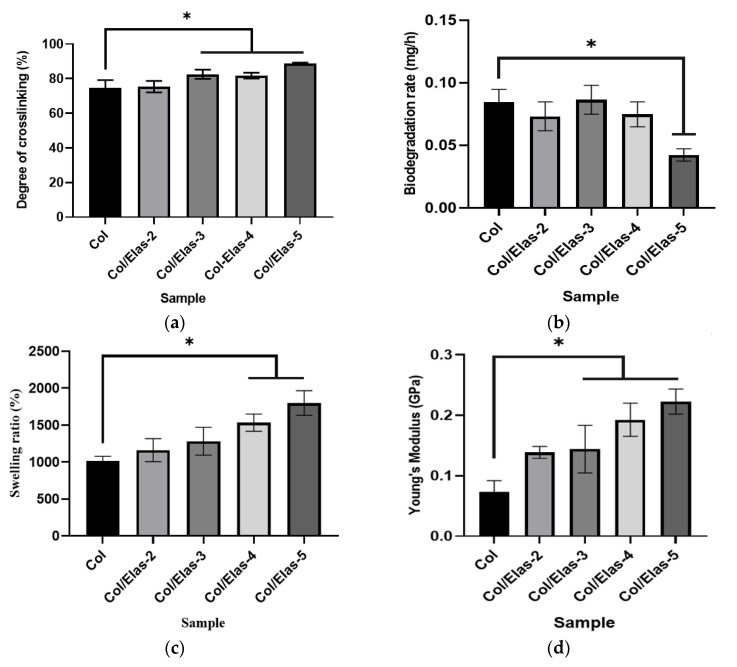
(**a**) The degree of crosslinking of Col/Elas scaffolds crosslinked by 0.1% genipin. (**b**) The enzymatic degradation of Col/Elas scaffolds in 0.0006% collagenase at 37 °C. (**c**) The water absorption capacity (%) of Col/Elas scaffolds. (**d**) Young’s modulus of Col/Elas scaffolds. (*) represents a significant difference (*p* < 0.05; *n* = 3) between fabricated scaffolds.

Figure 2a shows the degree of crosslinking of the scaffolds. This analysis was done to determine the crosslinking effectiveness of each sample, as assessed via a ninhydrin assay. The results showed that the degree of crosslinking was more than 80% and significantly increased for Col/Elas-3, Col/Elas-4 and Col/Elas-5. This indicates effective crosslinking when compared to Col (75%). The biodegradation rates of the scaffolds, which were determined via an enzymatic approach, are presented in Figure 2b. The biodegradation rate of Col/Elas-5 (0.04 ± 0.005 mg/h) was the slowest (*p* < 0.05) as compared to those of the other scaffolds. It is predicted that 10 mg of Col/Elas-5 scaffold will be fully degraded eight days after implantation.

The swelling ratio, or water absorption capacity, of the Col/Elas scaffolds is shown in Figure 2c. The swelling ratio for all samples was more than 1200%. The water absorption capacity increased significantly with the addition of elastin 4–5% in treated groups compared to Col. The Col/Elas-5 exhibited the highest water absorption of 1799 ± 167%.

The results of Young’s modulus are shown in Figure 2d. Scaffolds with a 5% addition of elastin had the highest values of the Young’s modulus. Col/Elas-5 had over three times the Young’s modulus value of the Col scaffold (0.22 vs. 0.07 GPa for the Col/Elas-5 and Col scaffolds, respectively).

### 3.2. Chemical Characterisation of Hybrid Bioscaffold

EDX was conducted to detect the elements that existed in the samples. The identified elements were carbon (C), oxygen (O) and nitrogen (N), which are the elements of the amide functional groups of both collagen and elastin (NH, C=O), as shown in Table 1. Carbon showed the highest percentage of atoms compared to other elements due to its abundance. The addition of elastin decreased the percentages of C and N, but it increased the percentage of O.

**Figure 3:**
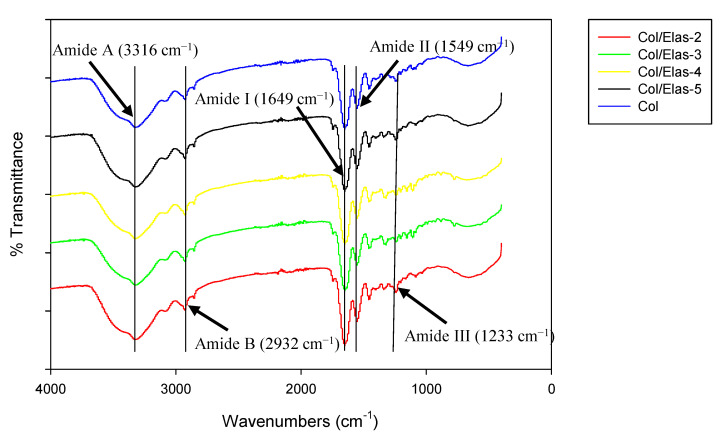
The FTIR spectra of Col/Elas scaffolds crosslinked by 0.1% genipin.

The FTIR spectra are presented in Figure 3: they showed typical spectra for proteins consisting of amides A, B, I, II and III. The prominent peaks of the amide I and amide II bands caused by the protein structures of each scaffold, were common to all samples. The stretching bonds of C=O corresponded with amide I bands. For the examined samples, these bands appeared at a wavelength of about 1649 cm^−1^. Amide II bands were connected to coupled N-H bending bonds and a C-N stretching bond. These bands occurred at about 1549 cm^−1^. Both collagen and elastin from the porcine aorta have similar bands of amide I (approximately 1580–1718 cm^−1^) and amide II (approximately 1480–1580 cm^−1^) absorbances [26,27]. Amide A bands, which represent stretching N-H bonds, occurred at approximately 3316 cm^−1^. The stretching vibrations of N-H in the Fermi resonance, which occurred at around 2932 cm^−1^, formed amide B bands. Another band was observed at a peak of 1233 cm^−1^ for amide III from the CH_2_ wagging vibration of proline side chains, which could have originated from the similar proline content in collagen and elastin [26,28].

### 3.3. Biocompatibility of Elas/Col Scaffolds

**Figure 4:**
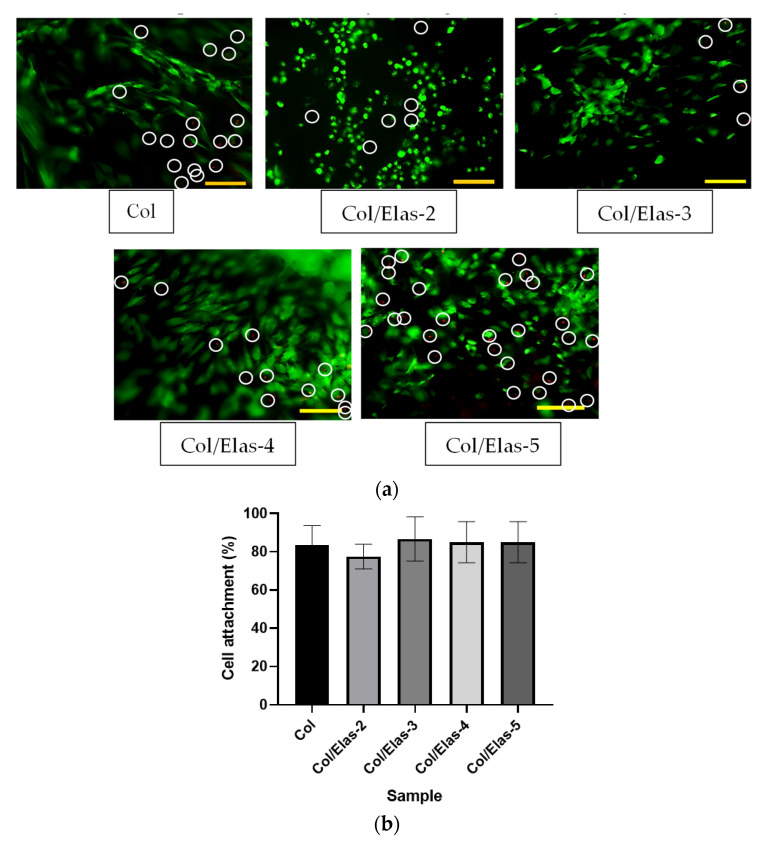

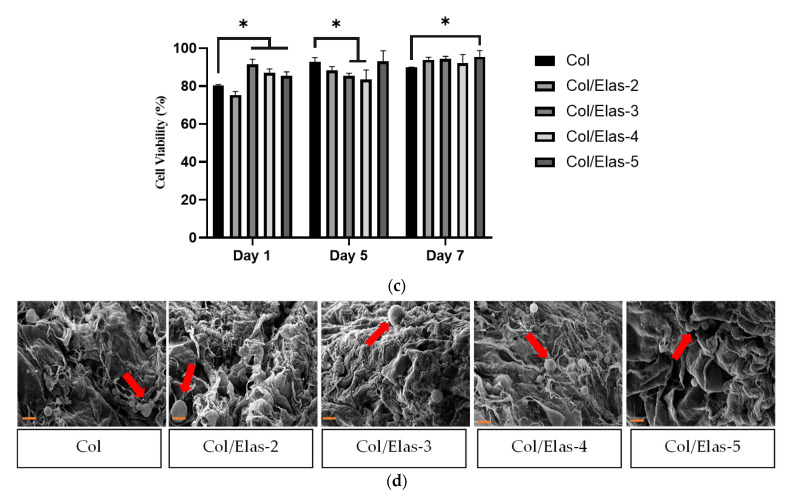
Cell-scaffold interaction in 3D scaffolds. (**a**) Live/Dead assay of the fabricated scaffolds; Scale bar: 100 µm. (**b**) The quantification of cell attachment at 24 h. (**c**) Cell viability of HDF on the scaffolds at day 1, 5 and 7 quantified by MTT assay. (**d**) Cross-sectional morphological features of HDF at 500× magnification; Scale bar: 20 µm. (*) represents a significant difference (*p* < 0.05; *n* = 3) between fabricated scaffolds.

It was found that HDF cultured on all scaffolds were viable (Figure 4a), as the presence of red HDF on scaffolds were few (white circles), and green HDF predominated, indicating no cytotoxic effect. The percentages of dead dells for Col, Col/Elas-2, Col/Elas-3, Col/Elas-4 and Col/Elas-5 were 16.2%, 1.6%, 3.5%, 9.2% and 16.6%, respectively. In comparison to the other samples, the cells on Col/Elas-2 had a round shape and less spindles. The samples showed good cell attachment within 24 h, where more than 80% of the cells successfully adhered onto the scaffolds as shown in Figure 4b. However, no differences in HDF attachment were observed between the Col scaffold and the other scaffolds. The FESEM images of the HDF morphologies on the fabricated scaffolds are shown in Figure 4d. Briefly, all scaffolds promoted cell adhesion and appeared round that had an average diameter of 20 μm with a surrounding matrix. They were found inside the scaffold, indicating that the cells migrated and attached successfully. Further evaluation was performed on the cell viability of HDF scaffolds using MTT assays at 1, 5 and 7 days (Figure 4c). Generally, each scaffold showed increased cell viability from day 1 to day 7. However, the elastin-based scaffolds (Col/Elas-3 and Col/Elas-4) showed less cell viability on day 5 as compared to Col, but they had higher viability on day 7.

## 4. Discussion

Multifunctional smart biomaterial is currently being explored in tissue engineering as an advanced technology that promotes the regeneration of skin that resembles native skin. However, when creating or establishing the suitability of a scaffold for use in tissue engineering, several crucial factors must be considered, including biocompatibility, biodegradability, mechanical properties, porosity and swelling ratio, all of which support tissue regeneration [29,30].

Thus, the aim of this study was to fabricate hybrid scaffolds containing elastin and collagen in order to evaluate the effect of adding elastin on the scaffold’s performance in optimising healing capacity while maintaining a better overall appearance. Gross appearance was examined to evaluate the physical appearance of the fabricated scaffolds. The results showed that the addition of 2–5% of elastin powder did not change the colour of the scaffold when compared to a Col sample. All samples underwent post-crosslinking with 0.1% (w/v) genipin to alter the final structural, mechanical and biological characteristics of the scaffolds. A natural crosslinker, genipin is made from the fruits of the *Gardenia jasminoides* plant. It is non-toxic and degrades more slowly than chemical crosslinkers such as glutaraldehyde [31,32]. Some size shrinkage was observed after the scaffolds were crosslinked. Crosslinking causes the samples to form intra- and intermolecular crosslinking networks with the free amino groups of the samples [33], thereby leading to size shrinkages.

In addition, both collagen and elastin are proteins; therefore, increasing the elastin concentration may form additional substrate for the crosslinking process and increase the crosslinking efficiency of the scaffolds [34]. Both the temperature and genipin concentration used during crosslinking are crucial factors that can affect the efficiency of crosslinking. It was discovered that the ideal genipin concentration for a gelatine scaffold is 0.5%, and that the ideal crosslinking temperature is 25 °C [32]. In this study, a slightly lower concentration of genipin (0.1%) but the same incubation temperature were used. Therefore, further studies could vary the genipin concentrations and time of incubation to improve the degree of crosslinking to more than 90%. In addition, crosslinking also affects swelling and degradation, hence the release of integrated bioactive chemicals from scaffolds. Therefore, having optimum crosslinking while fabricating biomaterials is crucial.

To maintain a moist environment, an optimal biomaterial in cutaneous wound healing must have a high-water absorption capacity. In this study, the fabricated hybrid scaffolds had good water absorption ability, which aids in the prevention of wound exudate accumulation in chronic skin wounds and the scaffolds expand upon contact with the wound. In addition to that, moist wounds are known to heal faster with an adequate supply of growth factors and other molecules to the healing tissues [35]. A moist environment also aids in providing essential nutrients to cells [35,36,37]. Compared to a dry environment, a moist environment speeds up the time a wound takes to heal because it makes it easier for growth factors to be released and for cells to multiply. It also helps basal epidermal cells to move into the wound. In addition, a moist wound environment minimises the risk of infection by promoting angiogenesis, lowering pH and making the wound unfriendly to bacteria [38].

The presence of hydroxyl (OH) and amino functional groups (NH_2_) of collagen and elastin, which can interact with water via hydrogen bonds, may explain the high swelling tendency of these scaffolds. Generally, the more crosslinking, the less swelling that is attainable [39,40]. However, these swelling results contradicted the usual notion that a higher degree of crosslinking leads to a lower swelling ratio. The presence of elastin increased the hydrophilic groups of the scaffolds, increasing their swelling ratio [41]. It is well known that collagen has good absorption properties that help to keep wounds hydrated [42]. Therefore, a combination of elastin and collagen could be useful for wound healing. In addition, the porous structure of scaffolds containing elastin allows fabricated scaffolds to entrap more water.

In the current study, all samples showed porosity of more than 60%, which is within the range of an ideal wound dressing requirement of 60–90% porosity [43]. The good porous structure of the fabricated scaffolds indicates that the freeze-drying method was both appropriate and the simplest approach for the fabrication of 3D scaffolds in this study. The collagen/elastin scaffolds cool to the freezing point during the freeze-drying process used to create these scaffolds, whereupon the ice phase separates to create a porous structure [44]. Specifically, Col scaffolds displayed a more compact, sheet-like microstructure, while treatment groups with elastin showed a fibre-like structures.

Mass transport, cell migration and tissue ingrowth into the implant’s structure are all greatly influenced by a scaffolds’ porosity, pore size and connection types [45]. The ideal pore diameter for scaffolds is thought to be between 100 and 500 µm [46]. Additionally, pores between 90 and 130 µm are the ideal size for skin regeneration and are sufficient to allow fibroblast migration and proliferation [47,48]. The collagen/elastin scaffold has good potential for use as a skin substitute due to its interconnected pores that are crucial for tissue repair, as well as having the right porosity and pore size.

In acellular skin substitutes, the biodegradation process is critical. Frequent dressing changes are not cost-effective and could be a burden to both patients and caregivers. Rapid degradation of a scaffold can impair its mechanical properties, while delayed degradation can cause an inflammatory reaction and lead to the failure of skin implantation [48]. The ideal scaffold would reduce inflammation, release medication and aid tissue regeneration without requiring frequent replacement. Since a scaffold is just a template and not permanent, it must be fully biodegradable to allow cells to produce their own ECM during the healing process [49]. In this study, the addition of elastin decreased the degradation rate of the scaffolds. A study done by Su, Fujiwara and Bumgardner [50] also showed that scaffolds with a high percentage of elastin had a slower degradation rate as compared to those with a low percentage. In addition, the degradation results showed that highly crosslinked samples will degrade more slowly compared with less crosslinked samples. This result shows that crosslinking can also improve the resistance of scaffolds against enzymatic degradation [44,50], thus enhancing the property of the scaffold as a skin substitute.

Pure collagen scaffolds have been shown to have poor mechanical characteristics. A scaffold needs to be strong enough to allow for surgical handling during implantation. To address this limitation, intermolecular crosslinking was attempted to enhance the mechanical strength and stability of the collagen scaffold [51]. The addition of elastin increases Young’s modulus’ values, which are indicative of the elasticity and rigidity of the samples [52]. In addition, the porosity of a scaffold is an important factor in influencing its mechanical properties. In this study, the addition of 2–3% elastin increased both the porosity of the scaffolds and their mechanical strength. Moreover, the addition of genipin makes the scaffolds more rigid without changing their porosity; it also has low toxicity [53]. In a study by Skopinska-Wisniewska et al. [27], the same pattern was observed in a high-compression modulus in relation to all of the crosslinked collagen–elastin-based samples.

The FTIR spectra obtained in this study revealed no changes in molecular structure after adding a mixture of collagen and elastin at various concentrations. This observation was also supported in earlier studies [26,27,54], which suggested that the addition of elastin and a crosslinking agent does not significantly affect collagen structure. Thus, it appeared that a hybrid scaffold was successfully fabricated while still maintaining the original characteristics from the functional groups.

Another consideration for an ideal scaffold for treating skin wounds is cellular compatibility, which can help human skin cells proliferate and remain viable. The results of a Live/Dead assay demonstrated that fibroblasts had excellent viability when adhering to and growing on the surfaces of collagen and elastin scaffolds. This implied that these scaffolds were not toxic to cells and were in fact inducing cell growth. A study on aortic elastin and collagen scaffolds also showed that fibroblasts adhered to and proliferated on scaffold surfaces with excellent cell viability [55]. Even though some dead cells were observed in this study, the number of dead cells was low compared to that of live cells. Furthermore, the round-shaped cells observed on Col/Elas-2 in the Live/Dead assay suggested that the cells were not fully proliferating yet, as evidenced by both the cell viability and cell attachment results, where Col/Elas-2 viability and attachment were low after 24 h. While there was some reduction in cell viability on day 5 for Col/Elas-3 and Col/Elas-4, it was not significantly different from day 1. When compared to Col, however, both Col/Elas-3 and Col/Elas-4 demonstrated significantly lower viability, most likely due to low cell proliferation rates and the need for time to become compatible with the scaffolds before accelerating proliferation as observed on day 7 [56]. Additionally, it has been demonstrated that combining elastin with collagen and gelatine encourages fibroblast proliferation in vitro [57]. All the abovementioned properties could lead to the better compatibility and attachment of the scaffolds.

## 5. Conclusions

In this study, a collagen/elastin scaffold was successfully fabricated using collagen from sheep and elastin from poultry, which is a low-cost, abundant and renewable material. This study presented the effects of collagen/elastin on the physicochemical and mechanical properties of the scaffold. In addition to its large surface area (due to a well-connected pore network structure), the collagen scaffold with elastin exhibited higher crosslinking efficiency (from 74.70% to 88.77%), higher water absorption (from 1012.70% to 1799.06%), lower degradation rates (from 0.085 mg/h to 0.043 mg/h) and higher mechanical strength (from 0.07 GPa to 0.22 GPa) than the Col scaffold. In addition, the hybrid Col/Elas scaffold exhibited good biocompatibility with all functional groups of the individual constituents (elastin and collagen) remaining intact and homogenous, as observed from the SEM, FTIR and EDX results. In summary, this study demonstrated the potential of combining collagen from ovine and poultry sources to create a hybrid scaffold with biomedical applications as an acellular skin substitute. However, further in vivo studies should be performed to better understand the potential of fabricated scaffolds for wound healing.

## Figures and Tables

**Table 1:** Elemental analysis by EDX in Col/Elas scaffolds. (*) represents a significant difference (*p* < 0.05; *n* = 3) from the control group, Col.

Sample	Element Weight (%)		
	C	O	N
Col	75.51 ± 2.48	23.37 ± 0.69	8.56 ± 0.83
Col/Elas-2	70.66 ± 2.89 *	23.79 ± 0.65	6.70 ± 0.62 *
Col/Elas-3	64.91 ± 2.07 *	29.06 ± 0.60 *	7.04 ± 0.18 *
Col/Elas-4	72.50 ± 2.00	24.07 ± 0.99	6.02 ± 0.20 *
Col/Elas-5	59.06 ± 1.36 *	32.93 ± 0.98 *	7.09 ± 0.69

## Data Availability

Not applicable.
